# A study on promoting AI learning and usage behaviors among health management students from the perspective of the “knowledge-belief-action” model

**DOI:** 10.3389/fpubh.2026.1747362

**Published:** 2026-04-22

**Authors:** Jinsong Du, Le Zhu, Xiaoqiang Min, Xiao Chang, Wenhao Qi, Tingting Wei, Shujie Wei, Xiaoyan Zhang, Jingya Huang, Xinru Tao, Hailing Zhou

**Affiliations:** 1 School of Health Management, Zaozhuang University, Zaozhuang, China; 2 Department of Geriatics, Shandong Healthcare Group Xinwen Central Hospital, Taian, China; 3 School of Public Administration, Hangzhou Normal University, Hangzhou, China; 4 School of Public Health and Nursing, Hangzhou Normal University, Hangzhou, China; 5 Department of Laboratory, Zaozhuang Municipal Hospital, Zaozhuang, China; 6 Image Center, Zaozhuang Municipal Hospital, Zaozhuang, China; 7 Department of Magnetic Resonance Imaging, Shandong Healthcare Group Zaozhuang Central Hospital, Zaozhuang, China

**Keywords:** AI, behavior promotion, college students, health management, knowledge-belief-action, machine learning

## Abstract

**Introduction:**

The application of Artificial Intelligence (AI) in the field of health management is becoming increasingly widespread, driving health services toward precision, personalization, and intelligence. This study aims to explore the behavior patterns of health management college students in AI learning and device usage. In addition, the study also aims to enhance students’ motivation and ability to apply AI by constructing a feedback oriented behavior promotion system based on machine learning technology.

**Methods:**

This study targeted college students from the School of Health Management at Zaozhuang University. A survey questionnaire based on the “Knowledge-Belief-Action” (KBA) model was designed, and a total of 184 valid responses were collected. The XGBoost algorithm was used to construct a predictive model for AI learning and usage behavior, and SHAP technology was applied for interpretative analysis of the model results to identify key influencing factors. Furthermore, the model was integrated into a web platform, and a visualized behavior promotion system was developed.

**Results:**

The accuracy, precision, recall, and F1-score of the predictive model all exceeded 0.698, indicating strong predictive capability. SHAP analysis revealed that factors such as knowledge mastery, awareness of ethical issues, and educational background have a significant impact on students’ AI learning and usage behavior. The behavior promotion system developed based on this model not only predicts students’ learning and usage behaviors but also provides a basis for personalized intervention.

**Discussion:**

This study combines the “KBA” model with machine learning to construct an interpretable predictive model for students’ AI learning and usage behavior. The study shows that ethical awareness, educational background, and practical application experience are important factors influencing students’ behavior. Based on this model, we further developed a behavior promotion system, providing new ideas and tools for optimizing AI education in universities. However, this study also has limitations, such as a single source of sample data. Future research could expand the sample range to further verify the generalizability of the research conclusions.

## 1 Introduction

Artificial Intelligence (AI) refers to the simulation, extension, and enhancement of human intelligence, enabling machines to perform tasks that typically require human intellect (1–3). In the field of health management, AI has demonstrated immense potential, driving health services toward more precise, personalized, and intelligent approaches (4, 5). In China, graduates of health management programs typically engage in community health monitoring, health education, and health guidance. In the context of digital transformation, the proficiency of students in utilizing large language models and other generative AI for efficient health education, or leveraging intelligent devices for precise risk assessment, has become a key determinant of their future professional competency and service effectiveness. Therefore, understanding health management students’ attitudes toward AI applications and the factors influencing these attitudes is crucial for cultivating highly qualified professionals who are capable of mastering advanced technologies and adapting to future health management needs.

The “Knowledge-Belief-Action” (KBA) model is a classic health behavior theory that emphasizes the gradual progression and interaction between individuals’ knowledge acquisition, belief formation, and behavior practice. It has been widely applied in health intervention research and other fields (6–8). For instance, Hasan et al. explored the knowledge, attitudes, and applications of AI in pharmacy practice among pharmacy students and faculty, showing a significant positive correlation between their attitudes toward AI and their usage behaviors (9). Other researchers such as Abou et al. also assessed the knowledge, attitudes, and skills of university nursing students regarding digital transformation, health literacy, and AI, and found that innovative undergraduate courses should be incorporated into the curriculum with practical opportunities in digital healthcare technology to improve students’ digital literacy and skills (10). These studies demonstrate that applying the KBA model to AI education can systematically reveal students’ behavioral mechanisms in AI learning and usage from the dimensions of knowledge, attitude, and behavior. This, in turn, helps universities accurately identify key points in educational content and design effective intervention strategies.

At the same time, with the rapid development of big data and machine learning technologies, predictive models based on multidimensional feature data have been increasingly applied in areas such as educational assessment and learning behavior analysis. For example, Rees et al. used machine learning techniques to screen medical residency applicants and predict whether they would be admitted (11). Hooper et al. also applied machine learning to predict whether students could meet the requirements for a Doctor of Veterinary Medicine degree, finding that GPA and economic factors were crucial determinants (12). These studies show that machine learning models can automatically extract effective information from complex variables, capturing nonlinear relationships and higher-order interactions that traditional statistical methods may overlook, thus enabling more accurate identification of high-risk groups. However, existing studies on predicting AI learning and usage behaviors primarily focus on model construction, lacking deep interpretations of feature importance within the models. Additionally, research findings are often not translated into practical application tools, limiting the operability and popularity of models in educational practices.

Against this background, this study targets college students from the School of Health Management as research subjects. Combining the KBA theory, a self-designed survey questionnaire was developed, covering three dimensions: knowledge, belief, and action. The XGBoost algorithm was employed to construct a predictive model for students’ AI learning and usage behaviors, and SHAP technology was introduced for interpretative analysis of the model results to identify key factors influencing these behaviors. Furthermore, the trained model and SHAP analysis results were integrated into a web platform to develop a visualized AI learning and usage behavior promotion system. This system creates a complete feedback loop from risk identification to personalized intervention recommendations, providing robust support for optimizing AI curriculum design and enhancing students’ comprehensive application skills in health management programs.

## 2 Methods

**Figure 1:**
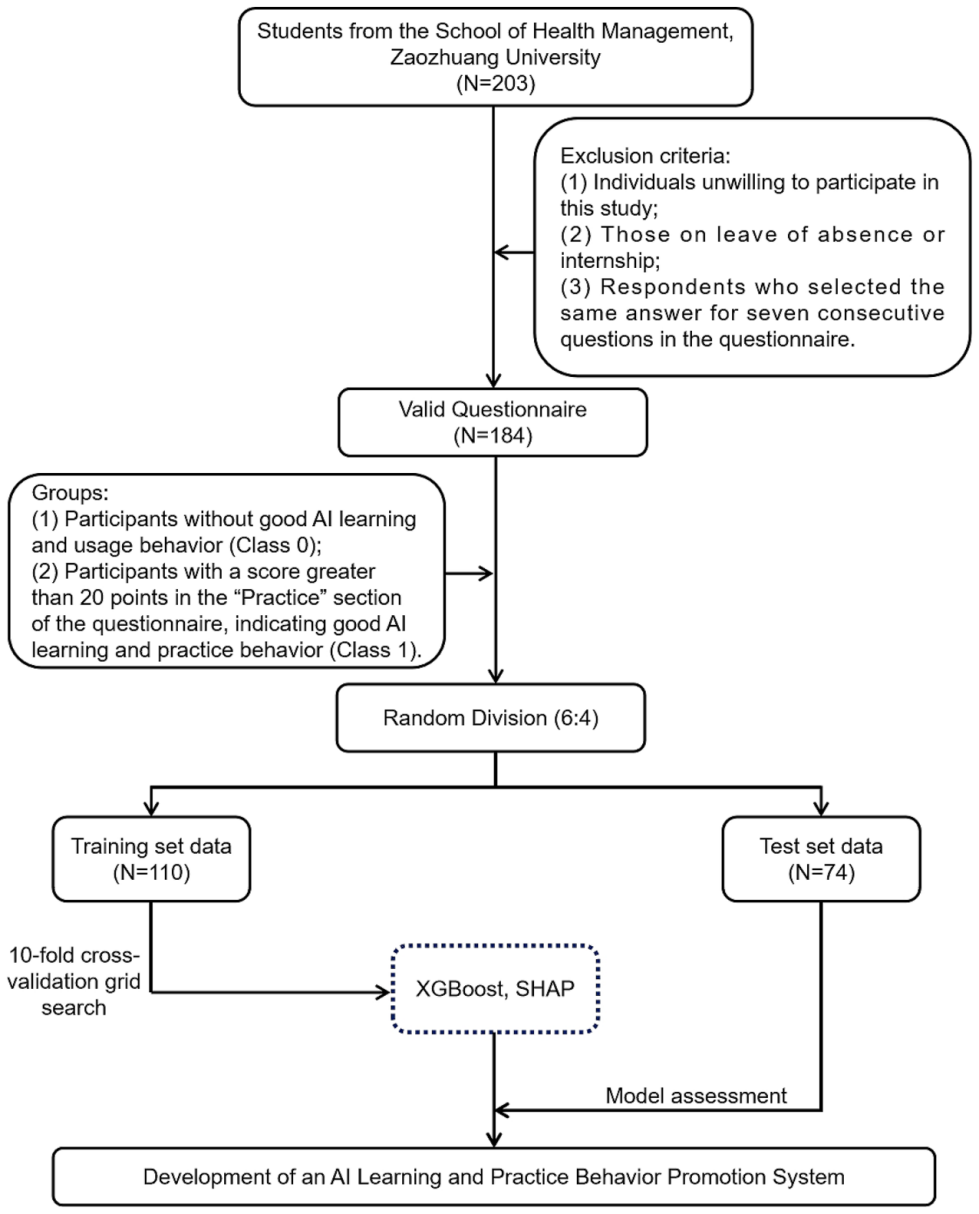
A flowchart describing the general framework of the study.

The research project has been approved by the Science and Ethics Committee of Zaozhuang University (Ethics Approval No.: 2025-PNSF-00102). Throughout the research process, the team adhered strictly to ethical standards and data protection principles, ensuring the legality, transparency, and scientific integrity of the study. This study was conducted in May 2025 at the School of Health Management, Zaozhuang University. The participants included first-year, second-year, and third-year students enrolled in the Undergraduate program in Health Services and Management and the Junior College program in Smart Health and Older Adult Care Services and Management (Figure 1). Data were collected using a combination of online and offline convenience sampling methods. The online questionnaires were distributed via the “Questionnaire Star” platform. The distribution and collection processes were uniformly administered by trained research assistants to ensure standardization and procedural consistency.

In the questionnaire instructions, participants were informed to complete the survey anonymously to control for experimenter and halo effects. The statement “there are no right or wrong answers” was clearly emphasized, and participants were assured that their responses would not influence academic evaluations, thereby minimizing the Hawthorne effect. Moreover, neutral wording and the principle of confidentiality were adopted to reduce the potential impact of self-report bias.

Exclusion criteria included: (1) individuals unwilling to participate in the study; (2) students who were on leave of absence or engaged in internships; (3) participants who selected the same answer for seven consecutive questions in the survey. A total of 203 questionnaires were distributed, and 184 valid questionnaires were returned.

The self-designed questionnaire (Supplementary Table S1) was based on the KBA model and contained 23 questions. Eight questions were demographic in nature and gathered basic information about the respondents. The remaining 15 questions assessed students’ knowledge, beliefs, and behaviors regarding basic AI concepts, data analysis, health education, smart health management devices, and ethical issues. For the KBA section, a 5-point Likert scale was used, with responses ranging from “Strongly Disagree” to “Strongly Agree,” assigned scores from 1 to 5. Following previous research (13), during the statistical processing stage, adjacent higher categories (scores of 4 and 5) on the Likert scale were regarded as “agree” responses to distinguish students with positive attitudes and behavioral performance toward AI learning and usage. Furthermore, students whose scores in the “Behavior” dimension exceeded 20 were defined as having good AI learning and usage behaviors.

A total of 203 questionnaires were distributed, and after excluding 19 invalid responses, 184 valid questionnaires were obtained. According to relevant methodological guidelines (14), the sample size should be at least five times the number of items, and the number of questionnaires distributed in this study meets this requirement. Reliability analysis results indicated high internal consistency for the questionnaire, with a Cronbach’s *α* = 0.896, demonstrating good reliability. Among the 184 valid respondents, 47 were male (25.6%) and 137 were female (74.4%), with ages ranging from 18 to 22 years (mean ± SD = 19.87 ± 0.98 years). In addition, 50 participants in this survey exhibited good AI learning and usage behaviors.

This study developed a behavior promotion system for college students’ AI learning and usage based on Python 3.11. First, the dataset was randomly split into training and testing sets at a 6:4 ratio, and the SMOTE algorithm was applied to address the data imbalance issue. The model utilized the XGBoost algorithm, with the optimal combination of hyperparameters determined through 10-fold cross-validation and grid search. Performance evaluation encompassed accuracy, precision, recall, F1-score, AUC values, and a confusion matrix, ensuring the comprehensiveness of the model assessment. To enhance the interpretability of the model, SHAP technology was introduced to calculate the importance index for each feature. Based on the analysis results, an online behavior promotion system integrating prediction, interpretation, and feedback was developed.

## 3 Results

**Figure 2:**
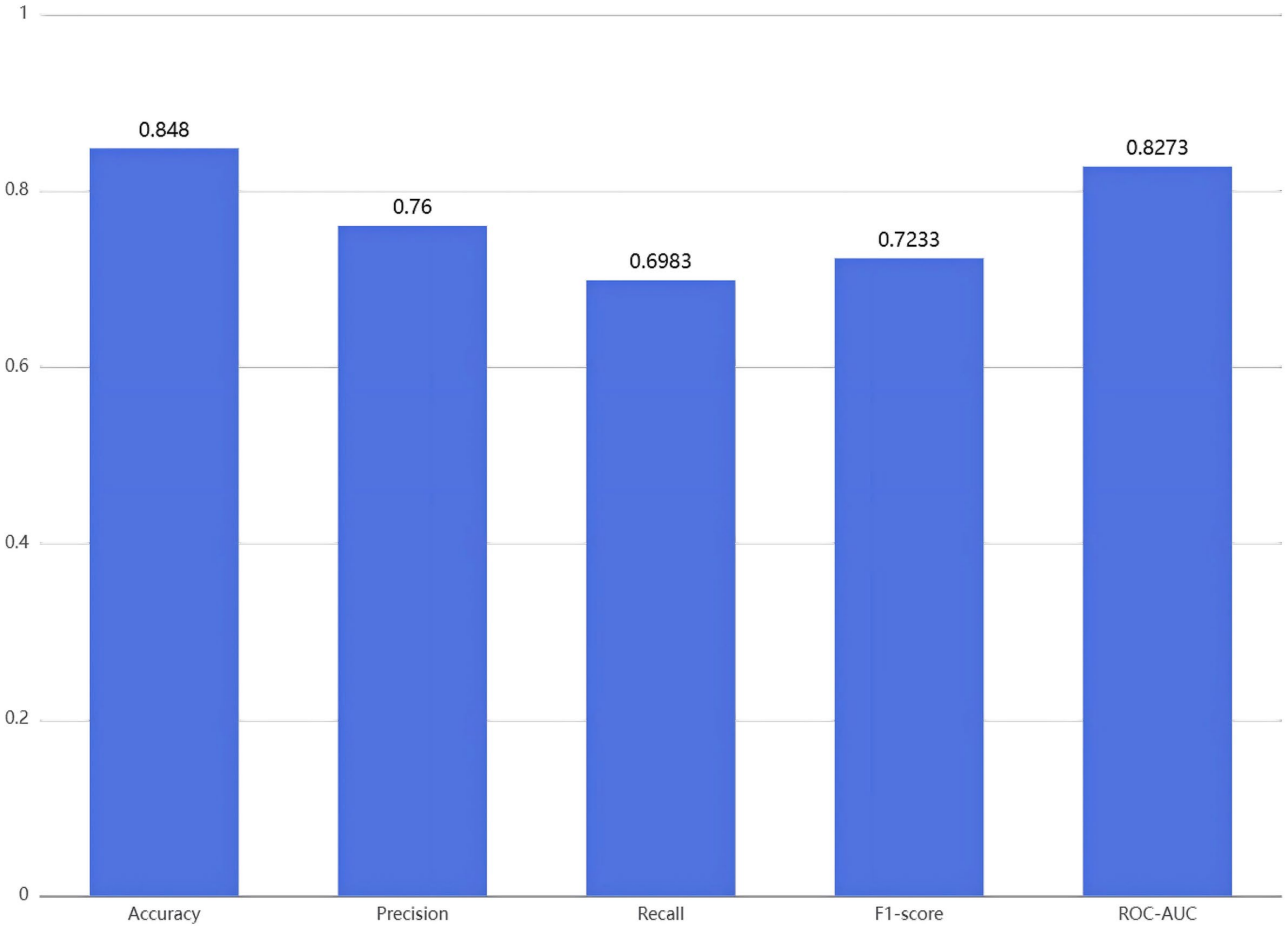
Mean accuracy, precision, recall, F1-score, and ROC-AUC of the XGB model across different random seeds.

The XGBoost algorithm was employed to construct the predictive model, and training and testing were repeatedly performed with different random seeds to evaluate the model’s robustness. The results showed that the average accuracy, precision, recall, and F1-score of the model all exceeded 0.698, with an average ROC-AUC value of 0.8273 (Figure 2), indicating strong predictive capability. Further analysis revealed that the fluctuation ranges of the ROC-AUC and F1-score were both within ±0.015 (Supplementary Table S2), suggesting that the model performed stably under different random partitioning conditions and demonstrated good robustness. This stability may be attributed to the efficiency and resilience of XGBoost in handling complex data structures, which enables it to effectively capture key features and suppress overfitting, thereby enhancing predictive performance (15, 16). In addition, the model’s precision was higher than its recall, indicating that it was more stable in identifying participants who did not exhibit good behaviors. This difference may be related to data imbalance, leading the model to be more inclined to predict the category of participants without good behavioral performance.

**Figure 3:**
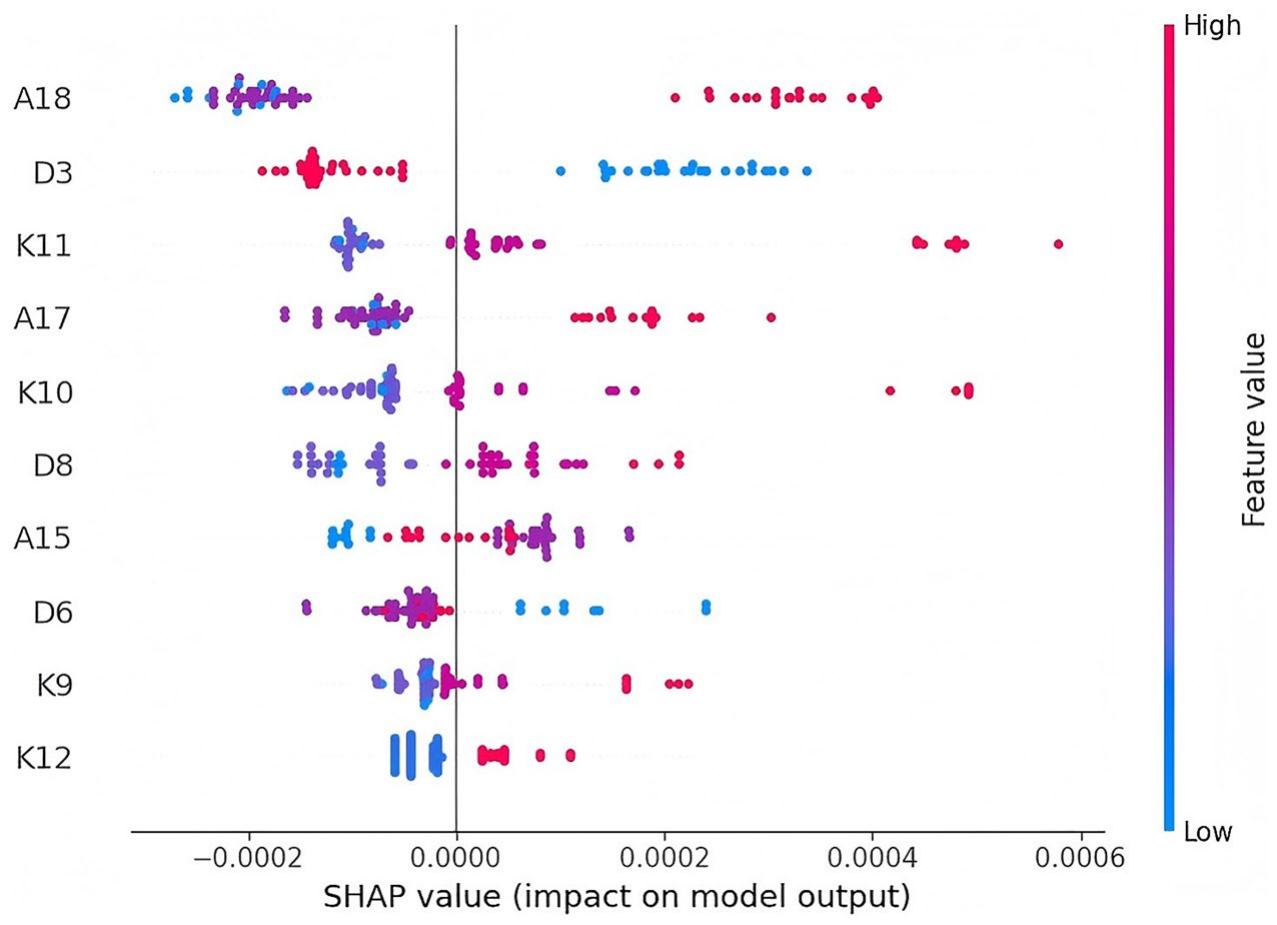
Analysis of the Feature Importance of Students’ AI Learning and Usage Behavior. (The detailed description of the features can be found in Supplementary Table S1).

SHAP plots are commonly used tools for visualizing and interpreting the predictions of machine learning models. They quantify the extent to which each input feature impacts the final prediction output (17, 18). By analyzing the SHAP values, we can determine whether a feature has a positive or negative influence on the prediction result and clearly illustrate the magnitude of this influence. In this study, we employed various SHAP visualization tools, including hexagonal plots and feature importance bar charts, to analyze the role of each variable in the model, explore the relationship between feature values and model outputs, and demonstrate the specific contributions of each feature in individual predictions. In the SHAP hexagonal plot, features are ranked from top to bottom according to their overall impact on the model’s prediction, and the horizontal axis displays the magnitude of each feature’s effect on the prediction. Each point represents a sample, and the color of the point reflects the value of the feature—red indicates high values, while blue indicates low values. This color coding allows for an intuitive visualization of the relationship between feature values and prediction probabilities. As shown in Figure 3, knowledge-related features such as “I understand how to use AI technology and devices for health education activities” and “I understand how to use AI technology for health data analysis and risk assessment,” belief-related features such as “I believe privacy protection, data security, and other ethical issues are prerequisites for using AI technology in health management” and “I believe smart health management devices such as rehabilitation robots can significantly improve the quality of daily health management,” and basic demographic features such as “Are you an undergraduate or associate degree student?” and “How interested are you in AI technology in health services?” all showed high importance in the model prediction. This indicates that these variables significantly influence whether students exhibit good AI learning and usage behaviors.

**Figure 4:**
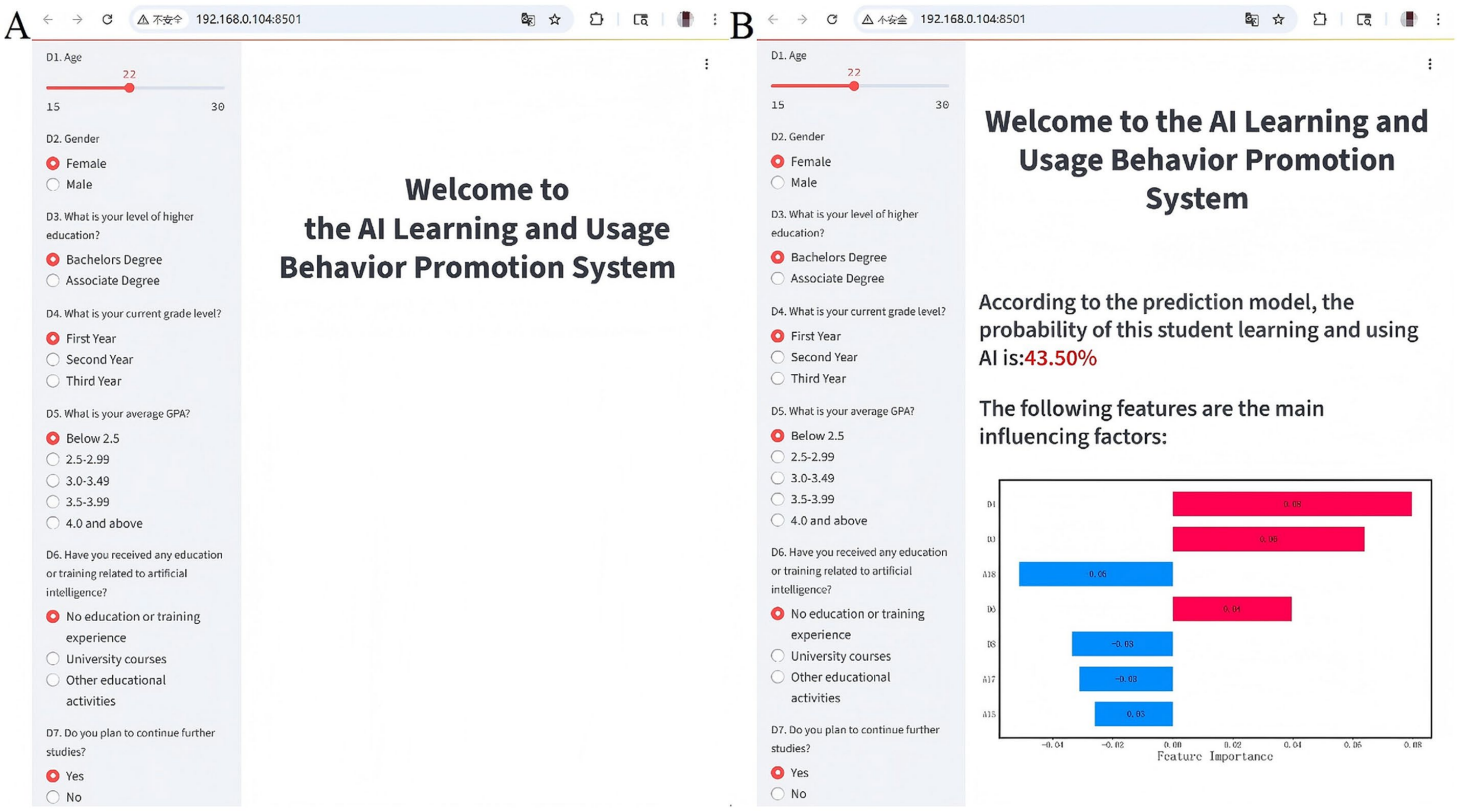
System for promoting students’ AI learning and usage behavior. **(A)** System homepage. **(B)** Information output page.

Based on the predictive model, we developed a student AI learning and usage behavior promotion system on a web platform (Figure 4). In this system, the left side of the interface is the information input area, where users can input personal information step-by-step according to the prompts. Continuous variables are adjusted using sliders, while categorical variables such as gender are selected by clicking options, ensuring ease of use and intuitiveness. The right side is the result display area, which shows the main influencing factors on users’ AI learning and usage behaviors, used for formulating behavior promotion strategies. Figure 4B illustrates an application example of the prediction system. After entering the relevant information on the left input interface, the SHAP plot on the right side of the system shows how each feature influences the prediction result. The length of the bars reflects the strength of the impact, with red bars indicating a positive influence and blue bars indicating a negative influence. Features that positively impact the prediction probability include D4, D3, and D6, while features such as A18, D8, A17, and A15 have a negative impact. Based on this analysis, targeted education on privacy ethics and device usage can be provided to users to improve their AI learning and usage behaviors.

## 4 Discussion

This study, based on the KBA model and machine learning algorithms, explored the impact of AI knowledge and attitudes on learning and usage behaviors, and constructed a behavior promotion system for AI learning and usage. The developed system not only predicts the probability of students’ engagement in AI learning and usage but also identifies and analyzes the key factors influencing their behaviors. Through web-based visual display, the system intuitively presents individual risk levels of AI learning and usage behaviors, the major influencing factors, and their relative importance. This establishes an information- and feedback-oriented behavioral support mechanism, providing teachers with intuitive decision-making support.

**Figure 5:**
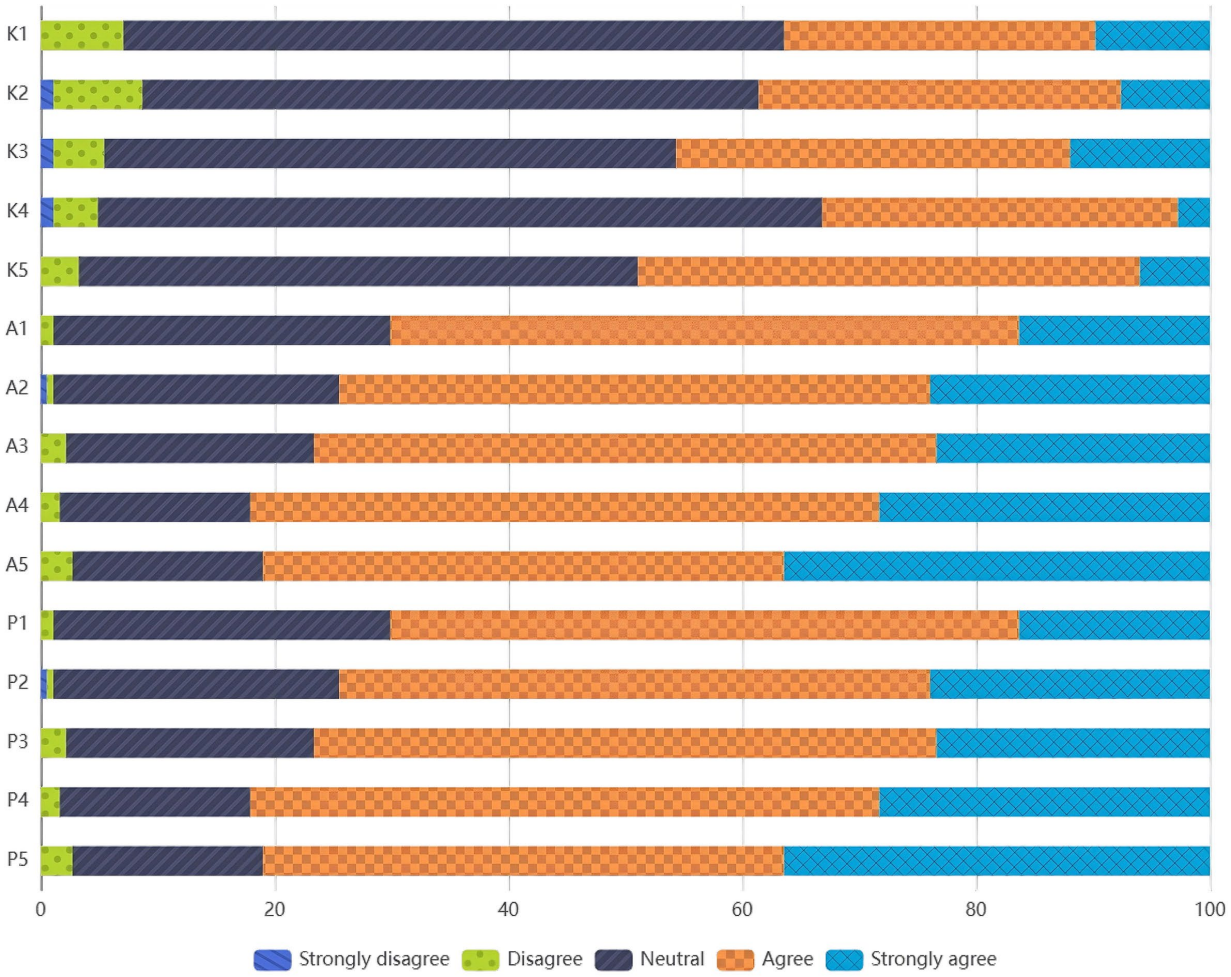
The distribution of respondents’ choices across the options.

Undoubtedly, AI plays an increasingly important role in current healthcare and health management. In this study, 57.1% of respondents expressed that they were “somewhat interested” or “very interested” in AI technology in health services (Figure 5). Similarly, Allam et al.’s study showed that 48% of Arab medical students displayed an open and positive attitude toward AI (19). Regarding health management students’ reception of AI education, 88.1% of the respondents indicated that they had “no education or training experience” or had received AI-related education or training through “university courses.” Neittaanmaki et al.’s study showed that 54% of Swedish medical students believed that AI should be part of medical training (20), Buabbas et al. found that 82.1% of Kuwaiti medical students thought they should receive AI education or training (21). These findings indicate that students are aware of the growing influence of AI in the healthcare industry, highlighting the importance of university education in promoting students’ mastery of AI knowledge and skills. AI is not merely a matter of simple tool operation; rather, it involves specific cognitive processes, such as digital health literacy, ethical judgment regarding medical AI, and a health management mindset characterized by human-machine collaboration. However, it must be acknowledged that health management students still face many challenges in learning and using AI, with only 36.41% of students “somewhat agreeing” or “strongly agreeing” that they understand the basic concepts and technologies of AI. Students’ understanding and mastery of AI knowledge and skills still need improvement, and concerns about ethics and security remain.

Based on the KBA model, this study employed the XGBoost algorithm to construct a predictive model for students’ AI learning and usage behaviors. The model performed well, with F1-score and ROC-AUC values above 0.698, demonstrating good predictive performance. Previous studies often focused on predicting student performance using demographic data. For example, Ojajuni et al. accurately predicted academic performance based on social and demographic information and historical data, helping educators identify and manage low-performing students (22). Asiksoy et al. classified online course performance based on emotional, cognitive, and behavioral factors, showing that features such as “expectations” were crucial to improving online learning outcomes (23). In contrast, this study followed the KBA theory to construct a questionnaire and analysis framework, with a focus on behavior prediction rather than performance prediction. This shift from “outcomes” to “processes” emphasizes dynamic recognition of the learning process, which helps identify key influencing factors of learning behaviors and provides a basis for tiered interventions and real-time support.

Building on the risk prediction model, we used SHAP to provide an interpretable visualization of the model. Since traditional machine learning methods often fail to clearly present the impact of features on decision-making, SHAP improves the interpretability of the results by quantifying and assigning the marginal contribution of each feature to the prediction outcome. Among all the feature variables, “I believe privacy protection, data security, and other ethical issues are prerequisites for applying AI technology in health management” (A18) had the most significant impact on the model’s predictions. From the perspective of the KBA model, this indicator represents the students’ “belief” dimension and ranks first in importance, strongly proving that the establishment of beliefs is the core driving force for learning behaviors and validating the applicability of the KBA theory within this group. This finding is consistent with the research of Abuadas et al., which suggests that students’ ethical awareness of AI is a significant influencing factor in predicting their usage behavior (24). Similarly, the study by Abou et al. indicated that ethical awareness and moral sensitivity are crucial for decision-making regarding the integration of AI technology in the healthcare field (10). This may be because a lack of moral sensitivity can lead students to feel ill-prepared when facing ethical challenges, thereby resulting in hesitation during practical application (25). This also suggests that in the context of health management, the establishment of beliefs is the core motivation for learning behaviors, reinforcing the applicability of the KBA theory for this population. Of course, the prominence of A18 may also be related to the single-source nature of the data in this study. The emergence of ethical awareness as a primary predictor in the model only represents the needs for ethics education among the research subjects in this study. Additionally, students’ education level (D3) is another important factor influencing the model’s predictions. This is consistent with Jiang et al.’s findings, which showed that undergraduate students and those in higher grades have higher interest and usage levels of AI (26). Furthermore, our study found that students who had previously used AI technology and devices for health education activities (K11) were more likely to continue learning and using AI. In conclusion, SHAP not only validated the model’s predictions but also revealed the key factors influencing students’ AI learning and usage behaviors. Based on this, we further deployed the predictive model to a web platform. The platform supports users in entering individual characteristic data, and based on SHAP analysis results, generates personalized reports on students’ AI learning and usage behavior. This helps to stimulate targeted learning motivation among students, facilitate personalized AI education in the field of health management, and assist students in achieving the leap from “knowledge” to “action.”

However, this study also has several limitations. First, regarding the sample dimension, the data were derived from a single institution and were characterized by an uneven gender distribution, which limits the generalizability of the conclusions to broader populations. In the design dimension, the cross-sectional data do not support causal inferences; future research should delve deeper using longitudinal studies. Second, this study did not propose an original AI implementation model at the algorithmic level but instead focused on empowering traditional KBA theory through the XGBoost-SHAP framework, providing interpretable predictive solutions and visualization tools for health management education. Future research could further enhance the theoretical depth and technical originality of the study by developing specific algorithms or introducing more situational variables. Additionally, although this study developed a behavior promotion system on a web-based platform, its primary function lies in assisting users to understand their own behavioral characteristics through risk visualization, thereby encouraging self-reflection and proactive adjustment. Future research will further explore the integration of automated educational prompts and intervention modules on this basis, aiming to construct a more comprehensive and closed-loop behavioral support system. Finally, although SHAP technology provided visual analysis of the model’s prediction results and revealed the contributions of different features, the specific ways in which some features influence the predictions have not been fully explained. Future research could explore these potential mechanisms further to deepen the understanding of students’ AI learning and usage behaviors.

## 5 Conclusion

This study developed a questionnaire on AI learning and usage behaviors for students of the School of Health Management based on the KBA model and used the XGBoost algorithm to construct a predictive model, which demonstrated good predictive ability. Through SHAP analysis, it was found that key factors influencing whether students exhibit good AI learning and usage behaviors include their level of AI-related knowledge, attention to ethical issues such as privacy protection and data security, education level, and practical experience in using AI technology and devices for health education. Based on these findings, the study further developed a web-based behavior promotion system, which can predict students’ learning and usage behaviors by inputting individual characteristics and present the main influencing factors in a visual format. This system provides intuitive, personalized intervention guidance for teachers and administrators. Overall, this study successfully developed a predictive model for AI learning and usage behaviors among students in health management programs, offering new ideas and tools for universities to conduct targeted AI education and enhance the knowledge and practical skills of health management students.

## Data Availability

The raw data supporting the conclusions of this article will be made available by the authors, without undue reservation.
