# Supplementary material for: A study on promoting AI learning and usage behaviors among health management students from the perspective of the “knowledge-belief-action” model
Source: Front Public Health. 2026 Apr 22;14:1747362. doi: 10.3389/fpubh.2026.1747362 (PMC13144107; doi:10.3389/fpubh.2026.1747362)
Supplement: Supplementary file 1 [file Data_Sheet_1.docx]

Supplementary Material

A Behavior Promotion System for AI Learning and Usage Among Health Management College Students Based on the “Knowledge Belief Action” Model and Machine Learning

**Supplementary Tables**

Table S1. Baseline Characteristics of the Study Population.

| **Variables** | **Distribution** |
| --- | --- |
| **Groups** | |
| **Class 0.** Participants without good AI learning and usage behavior. | 134 (72.83%) |
| **Class 1.** Indicating good AI learning and practice behavior. | 50 (27.17%) |
| **Demographic** | |
| **D1.** Age | 19.87 ± 0.98 |
| **D2.** Gender |  |
| Female (0) | 137 (74.46%) |
| Male (1) | 47 (25.54%) |
| **D3.** Higher Education Level |  |
| Undergraduate (1) | 78 (42.39%) |
| Junior College (2) | 106 (57.61%) |
| **D4.** Grade |  |
| First Year (1) | 49 (26.63%) |
| Second Year (2) | 109 (59.24%) |
| Third Year (3) | 26 (14.13%) |
| **D5.** Average GPA |  |
| Below 2.5 (1) | 0 (0.00%) |
| 2.5–2.99 (2) | 6 (3.26%) |
| 3.0–3.49 (3) | 65 (35.33%) |
| 3.5–3.99 (4) | 80 (43.48%) |
| 4.0 and above (5) | 33 (17.93%) |
| **D6.** Have you received any AI-related education or training? |  |
| No education or training experience (1) | 37 (20.11%) |
| University courses (2) | 124 (67.39%) |
| Other educational activities (3) | 23 (12.50%) |
| **D7.** Do you plan to pursue further education (e.g., a postgraduate degree)? |  |
| Yes (1) | 75 (40.76%) |
| No (2) | 24 (13.04%) |
| Uncertain (3) | 85 (46.20%) |
| **D8.** How interested are you in AI technology in health services? |  |
| Very uninterested (1) | 3 (1.63%) |
| Somewhat uninterested (2) | 13 (7.07%) |
| Neutral (3) | 63 (34.24%) |
| Somewhat interested (4) | 86 (46.74%) |
| Very interested (5) | 19 (10.33%) |
| **Knowledge** | |
| **K1.** I understand the basic concepts and related technologies of artificial intelligence. |  |
| Strongly disagree (1) | 0 (0.00%) |
| Disagree (2) | 13 (7.07%) |
| Neutral (3) | 104 (56.52%) |
| Agree (4) | 49 (26.63%) |
| Strongly agree (5) | 18 (9.78%) |
| **K2.** I understand how to use AI technology for health data analysis and risk assessment. |  |
| Strongly disagree (1) | 2 (1.09%) |
| Disagree (2) | 14 (7.61%) |
| Neutral (3) | 97 (52.72%) |
| Agree (4) | 57 (30.98%) |
| Strongly agree (5) | 14 (7.61%) |
| **K3.** I understand how to apply AI technologies and devices in health education activities. |  |
| Strongly disagree (1) | 2 (1.09%) |
| Disagree (2) | 8 (4.35%) |
| Neutral (3) | 90 (48.91%) |
| Agree (4) | 62 (33.70%) |
| Strongly agree (5) | 22 (11.96%) |
| **K4.** I am familiar with AI-based health management devices such as smart mattresses, bio-radars, and rehabilitation robots. |  |
| Strongly disagree (1) | 2 (1.09%) |
| Disagree (2) | 7 (3.80%) |
| Neutral (3) | 114 (61.96%) |
| Agree (4) | 56 (30.43%) |
| Strongly agree (5) | 5 (2.72%) |
| **K5.** I understand ethical issues and challenges that may arise when using AI in health management. |  |
| Strongly disagree (1) | 0 (0.00%) |
| Disagree (2) | 6 (3.26%) |
| Neutral (3) | 88 (47.83%) |
| Agree (4) | 79 (42.93%) |
| Strongly agree (5) | 11 (5.98%) |
| **Attitude** | |
| **A1.** I believe that mastering AI concepts and technologies is fundamental for current health management services. |  |
| Strongly disagree (1) | 0 (0.00%) |
| Disagree (2) | 2 (1.09%) |
| Neutral (3) | 53 (28.80%) |
| Agree (4) | 99 (53.80%) |
| Strongly agree (5) | 30 (16.30%) |
| **A2.** I believe that the use of AI in health data analysis and risk assessment can significantly improve precision and efficiency in disease prevention and personalized treatment. |  |
| Strongly disagree (1) | 1 (0.54%) |
| Disagree (2) | 1 (0.54%) |
| Neutral (3) | 45 (24.46%) |
| Agree (4) | 93 (50.54%) |
| Strongly agree (5) | 44 (23.91%) |
| **A3.** I believe that AI technologies and devices can effectively enhance public participation and the dissemination of health knowledge in health education. |  |
| Strongly disagree (1) | 0 (0.00%) |
| Disagree (2) | 4 (2.17%) |
| Neutral (3) | 39 (21.20%) |
| Agree (4) | 98 (53.26%) |
| Strongly agree (5) | 43 (23.37%) |
| **A4.** I believe that smart health management devices, such as rehabilitation robots, can significantly improve the quality of daily health management. |  |
| Strongly disagree (1) | 0 (0.00%) |
| Disagree (2) | 3 (1.63%) |
| Neutral (3) | 30 (16.30%) |
| Agree (4) | 99 (53.80%) |
| Strongly agree (5) | 52 (28.26%) |
| **A5.** I believe that privacy protection and data security are prerequisites for applying AI technology in health management. |  |
| Strongly disagree (1) | 0 (0.00%) |
| Disagree (2) | 5 (2.72%) |
| Neutral (3) | 30 (16.30%) |
| Agree (4) | 82 (44.57%) |
| Strongly agree (5) | 67 (36.41%) |
| Practice | |
| **P1.** I will continue or proactively learn the basic concepts and technologies of AI in the future. |  |
| Strongly disagree (1) | 0 (0.00%) |
| Disagree (2) | 2 (1.09%) |
| Neutral (3) | 53 (28.80%) |
| Agree (4) | 99 (53.80%) |
| Strongly agree (5) | 30 (16.30%) |
| **P2.** I will continue or proactively learn how to use AI for health data analysis. |  |
| Strongly disagree (1) | 1 (0.54%) |
| Disagree (2) | 1 (0.54%) |
| Neutral (3) | 45 (24.46%) |
| Agree (4) | 93 (50.54%) |
| Strongly agree (5) | 44 (23.91%) |
| **P3.** I will continue or proactively apply AI technologies and devices in health education activities. |  |
| Strongly disagree (1) | 0 (0.00%) |
| Disagree (2) | 4 (2.17%) |
| Neutral (3) | 39 (21.20%) |
| Agree (4) | 98 (53.26%) |
| Strongly agree (5) | 43 (23.37%) |
| **P4.** I will continue or proactively learn and use AI-based health management devices such as rehabilitation robots. |  |
| Strongly disagree (1) | 0 (0.00%) |
| Disagree (2) | 3 (1.63%) |
| Neutral (3) | 30 (16.30%) |
| Agree (4) | 99 (53.80%) |
| Strongly agree (5) | 52 (28.26%) |
| **P5.** I will always adhere to ethical principles to ensure that AI applications in health management comply with moral and legal standards. |  |
| Strongly disagree (1) | 0 (0.00%) |
| Disagree (2) | 5 (2.72%) |
| Neutral (3) | 30 (16.30%) |
| Agree (4) | 82 (44.57%) |
| Strongly agree (5) | 67 (36.41%) |

Table S2. Accuracy, precision, recall, F1-score, and ROC-AUC of the XGB model under different random seeds.

| Random seed | Accuracy | Precision | Recall | F1-score | ROC-AUC |
| --- | --- | --- | --- | --- | --- |
| 1 | 0.8649 | 0.7 | 0.7778 | 0.7368 | 0.8204 |
| 2 | 0.8571 | 0.7333 | 0.7333 | 0.7333 | 0.8325 |
| 3 | 0.8571 | 0.7143 | 0.7143 | 0.7143 | 0.8214 |
| 4 | 0.8214 | 0.8667 | 0.6190 | 0.7222 | 0.8327 |
| 5 | 0.8393 | 0.7857 | 0.6471 | 0.7097 | 0.8296 |
| Mean | 0.8480 | 0.76 | 0.6983 | 0.7233 | 0.8273 |
